# Splice form variant and amino acid changes in MDR49 confers DDT resistance in transgenic *Drosophila*

**DOI:** 10.1038/srep23355

**Published:** 2016-03-22

**Authors:** Keon Mook Seong, Weilin Sun, John M. Clark, Barry R. Pittendrigh

**Affiliations:** 1 Department of Entomology, University of Illinois at Urbana-Champaign, Illinois, 61801, USA; 2 Department of Veterinary & Animal Science, University of Massachusetts, Amherst, MA, 01003, USA

## Abstract

The ATP-binding cassette (ABC) transporters represent a superfamily of proteins that have important physiological roles in both prokaryotes and eukaryotes. In insects, ABC transporters have previously been implicated in insecticide resistance. The *91-R* strain of *Drosophila melanogaster* has been intensely selected with DDT over six decades. A recent selective sweeps analysis of *91-R* implicated the potential role of *MDR49*, an ABC transporter, in DDT resistance, however, to date the details of how *MDR49* may play a role in resistance have not been elucidated. In this study, we investigated the impact of structural changes and an alternative splicing event in *MDR49* on DDT-resistance in *91-R*, as compared to the DDT susceptible strain *91-C*. We observed three amino acid differences in MDR49 when *91-R* was compared with *91-C*, and only one isoform (MDR49B) was implicated in DDT resistance. A transgenic *Drosophila* strain containing the *91-R*-*MDR49B* isoform had a significantly higher LD_50_ value as compared to the *91-C*-*MDR49B* isoform at the early time points (6 h to 12 h) during DDT exposure. Our data support the hypothesis that the *MDR49B* isoform, with three amino acid mutations, plays a role in the early aspects of DDT resistance in *91-R*.

Post World War II, many agriculturally or medically important pests have been managed through the application of second-generation insecticides. This form of selection pressure has led to the evolution of pesticide resistance in many of the target insect populations^1^. Insecticide resistance is a valuable model for studying molecular evolution, as it is a well-defined system of man-made selection using known insecticides^2^,^3^. One such insecticide is the neurotoxic organochlorine pesticide 4,4′-dichlorodiphenyltrichloroethane (DDT), which affects the arthropod nervous system by interfering with normal nerve impulses. The insecticidal properties of DDT were discovered by Paul Müller in 1939 and due to its widespread use and effectiveness, DDT went from being a panacea for insect control to being banned for use in most countries in the 1970s. Nevertheless, the use of DDT for vector control continues and may increase as insect-borne diseases expand^4^,^5^.

Resistance to DDT has been documented across many pest species along with the non-target species, such as *Drosophila melanogaster* (*Drosophila*)^3^,^5^. DDT resistance in *Drosophila* has been used for the study of the evolution of insecticide resistance^6^. Historically, two major mechanisms of DDT resistance in *Drosophila* have been reported. First, resistance has been associated with amino acid changes in the voltage-gated sodium channel resulting in channel insensitivity to DDT^7^. Second, metabolic resistance to DDT has been observed in field populations of *Drosophila*^8^. Enhanced xenobiotic metabolism is an important form of resistance and is associated with alterations (or some cases structural changes) in the activities or levels of detoxification enzymes, such as cytochrome P450s, glutathione-S-transferases (GSTs), esterases or a combination of these activities^8^,^9^,^10^,^11^. Furthermore, proteomics-based profiling identified abundant proteins associated with DDT resistance in field- and laboratory-selected resistant *Drosophila*^12^.

The DDT resistance phenotype in *Drosophila* is not uniform, resulting in varying levels of resistance observed across different *Drosophila* strains, and resistance can be generally categorized into low, medium and high levels as measured by lethal concentration 50 (LC_50_)^8^,^10^,^11^. One pair of *Drosophila* strains, which are notably important for the study of the high level DDT resistance phenotype, are *91-R* and its DDT susceptible counterpart *91-C*. The two strains originated from a common population^13^,^14^,^15^. The laboratory selected DDT resistance strain *91-R* has been exposed to prolonged and periodic artificial selection with DDT for 60 years and has thus become highly resistant to DDT whereas *91-C* has not been exposed to DDT selection.

In the *91-R* strain, constitutive over-expression of *Cyp12d1, Cyp6a2* and *Cyp6g1* have been observed^8^,^16^,^17^, however, a recent selective sweeps analysis between *91-R* and *91-C* demonstrated that thirteen major and three minor effect chromosome intervals, with reduced nucleotide diversity, were identified only in the *91-R* strain^18^. Interestingly, of these thirteen major and three minor loci, the only cytochrome P450 observed was *Cyp4g1*, which is thought to be associated with the reduced curricular penetration phenotype^19^. Another gene, multidrug resistance 49 (*MDR49*) was located in one of the other major effect chromosome intervals in the selective sweeps analysis.

Multidrug resistance genes are known to code for ATP-binding Cassette (ABC) transporter proteins. ABC transporters are ATP-dependent efflux pumps belonging to extensive family of transmembrane proteins. The ABC protein family is present in the cellular membrane in all organisms and mediate the efflux of a wide variety of substrates, including sugars, amino acids, lipids, and xenobiotics to the outside of the cell and these transporters prevent the accumulation of harmful toxicants inside the cells^20^,^21^. ABC transporters are structurally characterized by four functional units: two highly conserved nucleotide-binding domains (NBDs), which are responsible for ATP-binding and hydrolysis, providing the energy for active transporting substrates across cellular membrane; and, two highly hydrophobic transmembrane domains (TMDs), which are involved in physical pathway for substrate translocation. Unlike the NBDs, TMDs vary in sequence, length and helix number^22^.

Based on their sequence similarity, domain structures, and organization, ABC transporters can be subdivided into eight subfamilies, designated ABC-A to ABC-H. Insect ABC transporters have diverse functions that affect molting, metabolism, cuticle differentiation, and egg development^23^,^24^,^25^. They are also thought to be associated with defense or resistance to plant defensive compounds and numerous insecticides by reducing toxic concentrations in tissues^26^. Unlike the other subfamilies, the ABC-B, ABC-C, and ABC-G have been associated with drug resistance and detoxification in insect pests^26^,^27^. For example, involvement of ABC transporters in pyrethroid resistance has been reported in *Helicoverpa armigera*^28^, *Apis mellifera*^29^ and *Culex pipiens*^30^.

ABC transporters have also been implicated in DDT resistance in *Drosophila.* The ABC-B subfamily multiple drug resistance (*MDR*) genes, *MDR50, MDR65,* as well as the ABC-C subfamily multidrug resistance-associated protein gene, *MRP1,* are constitutively overexpressed in the DDT-resistant *91-R* strain when compared with the DDT-susceptible *Canton-S* strain. The ABC-B subfamily multiple drug resistance gene *MDR49,* however, was not overexpressed in DDT-resistant *91-R* strain^19^,^31^,^32^. Additionally, RNAi knockdown of *MDR50, MDR65*, and *MRP1*, using transgenic GAL4/UAS-RNAi flies, in conjunction with DDT bioassays, confirmed the potential role of these genes in DDT resistance/susceptibility. Interestingly, *MDR49*, which was implicated as a putative resistance locus in the selective sweeps analysis, was not over transcribed in *91-R* versus the *Canton-S* strain^31^,^32^. These combined observations lead us to test the hypothesis that *MDR49* may play a role in DDT resistance through structural changes in the gene (and the resultant proteins) as opposed to increased levels of expression.

In the present study, we focused on the ABC-B subfamily genes, *MDR49, MDR50*, and *MDR65*. Using RNA-seq data, we compared open reading frames of all three genes and alternative splicing in *MDR49*. As *MDR49* was the only *MDR* to be in a major effect chromosome interval in the selective sweeps analysis^18^, we also created transgenic *Drosophila* containing both alternative splice forms of *MDR49*, with the respective amino acid differences between *91-R* and *91-C,* to determine if structural differences in *MDR49* proteins, between *91-R* and *91-C*, may play a role in DDT resistance.

## Results

### Sequence differences in *MDR49, MDR50,* and *MDR65* between the *91-R* and *91-C* strains

We compared the sequences of the *MDR49, MDR50,* and *MDR65* genes between *91-R* and *91-C* to determine non-silent mutations within the ORFs that may lead to potential amino acid replacement. Using the GenBank database, two different transcript isoforms were observed from the *MDR49* gene, designated as *MDR49A* and *MDR49B*. Although the sequences of both transcript isoforms were completely identical, the first four exons were alternatively spliced out of *MDR49A,* giving rise to *MDR49B*. Several SNPs were detected when *91-R* was compared with *91-C*. There were 39 SNPs observed when the *MDR49*A isoform from *91-R* was compared with the *MDR49A* isoform from *91-C*. Similarly, there were 37 SNPs observed when the *MDR49B* isoform from *91-R* was compared with the *MDR49B* isoform from *91-C*. Most SNPs resulted in silent mutations, however, three SNPs resulted in non-silent mutations in both the *MDR49A* and *B* genes from *91-R* when compared with *91-C*.

**Figure 1:**
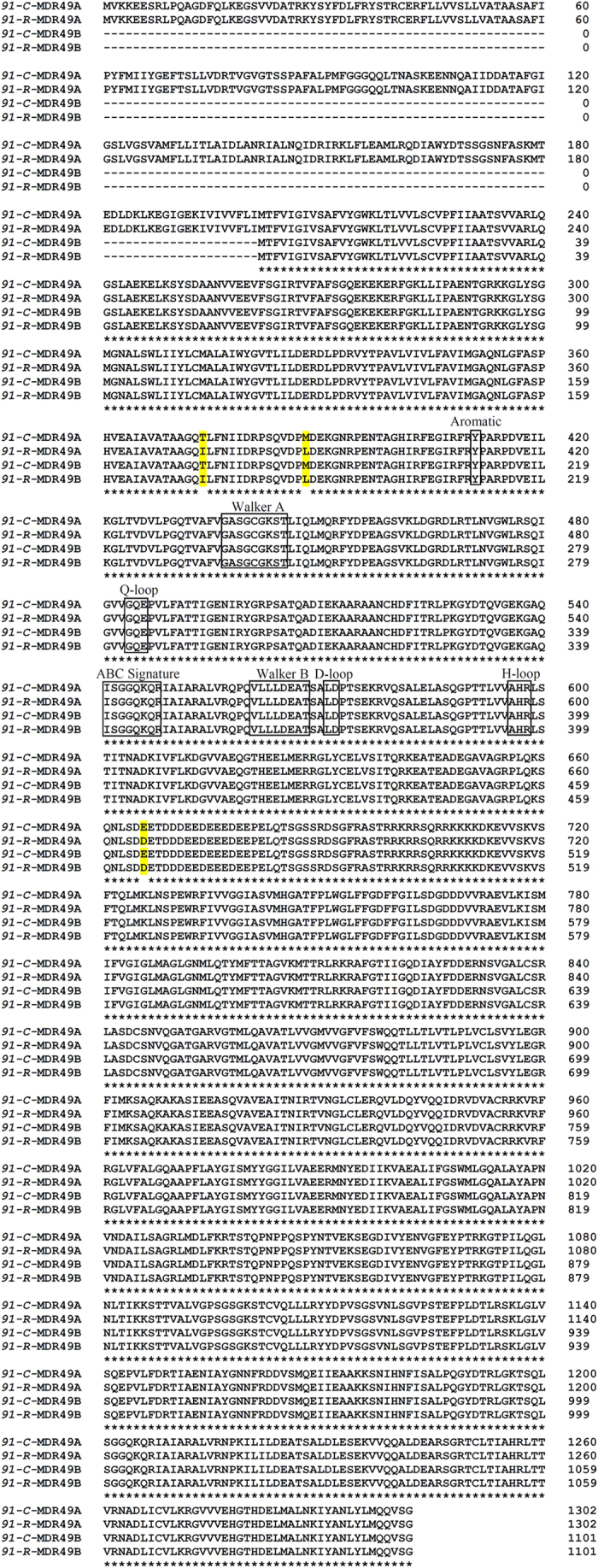
Sequence alignment of putative amino acids deduced from the MDR49 proteins associated with the *91-C* and *91-R* strains. Three mutations that resulted in amino acid replacements, T374I, M388L, and E666D for MDR49A; T173I, M187L, and E465D for MDR49B were observed in *91-R* when compared with *91-C*. The NBDs were homologous in both strains and each NBD had seven highly conserved motifs (aromatic, Walker A, Q-loop, ABC signature, Walker B, D-loop, and H-loop), which appear in boxes.

Thr374, Met388, and Glu666 in MDR49A protein from *91-C* were replaced with Ile374, Leu388, and Asp666 from *91-R*; Thr173, Met187, and Glu465 in MDR49B protein from *91-C* was substitute with Ile173, Leu187, and Asp465 from *91-R* (Fig. 1). *MDR50* and *MDR65* were also determined to have SNPs (4 and 17, respectively) when *91-R* was compared with *91-C*. These SNPs resulted in three non-silent mutations: Thr393, Ser881, and Val1012 in MDR50 protein from *91-C* was replaced with Ala393, Phe881, and Leu1012 in *91-R* (Supplementary Fig. S1); Lys277, Ile646, and Leu990 in MDR65 protein from *91-C* was substituted with Arg277, Met646, and Pro990 in *91-R* (Supplementary Fig. S2). Interestingly, a total of three non-silent mutations were detected in each of the three *MDR* genes from the *91-R* strain and each mutation was novel, suggesting that these residue changes likely cause structural alternation in these three MDR proteins from the DDT-resistant strain.

### Prediction of structure for three MDR proteins

**Figure 2:**
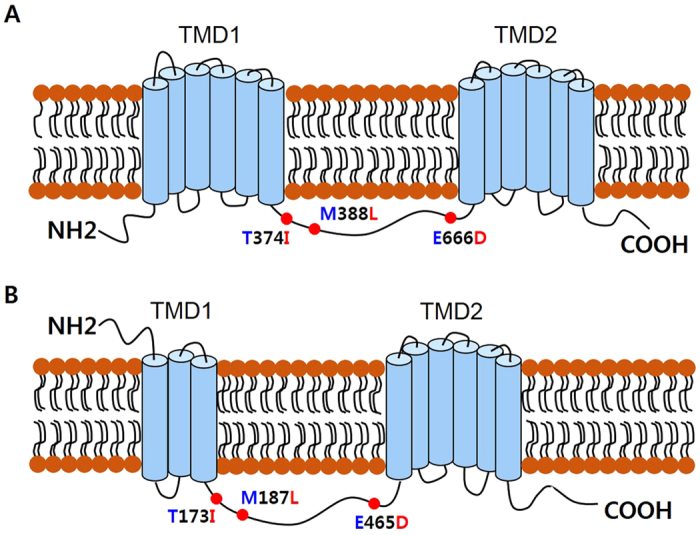
Schematic diagrams of the predicted structures of the MDR49 proteins, encompassing the T374I, M388L, and E666D for MDR49A; T173I, M187L, and E465D for MDR49B amino acid replacement sites. (**A**) Twelve transmembrane segments in two transmembrane domains (TMD 1 and TMD 2) were predicted in the MDR49A isoform. (**B**) Nine transmembrane segments were predicted in two TMDs in the MDR49B isoform. Amino acid replacements that may result in structural alterations are indicated by red dots. Blue letters represent *91-C*; red letters represent *91-R.*

**Figure 3:**
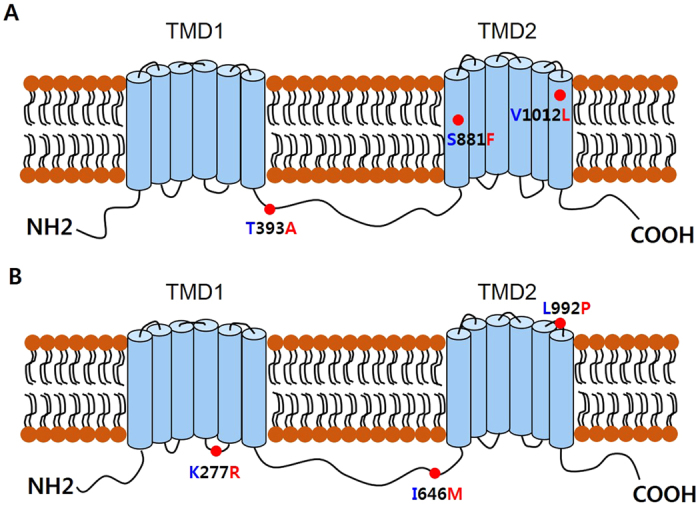
Schematic diagrams of the predicted structures of the MDR50 and MDR65 proteins, each encompassing twelve transmembrane segments in two TMDs. (**A**) The MDR50 protein had the T393A, S881F, and V1012L amino acid replacement sites. (**B**) The MDR65 protein had the K277R, I646M, and L992P sites. Amino acid replacements that may result in structural changes are indicated by red dots. Blue letters represent *91-C*; red letters represent *91-R.*

Using the NCBI CDS-Conserved-Domains-prediction-server, the structural features of each of the aforementioned MDR proteins were predicted, including the alternative splice forms for *MDR49* (discussed further in the next section). The deduced amino acid sequences for each of the three MDR proteins had characteristic features of ABC transporters, including one cytoplasmic N-terminus nucleotide binding domain (NBD) and several conserved motifs (Walker A motif, Walker B motif, and C-motif) (Fig. 1 and Supplementary Fig. S1, S2). The two alternative splice forms of MDR49 showed atypical variations in their transmembrane domains (TMDs), which were different from each other. For the MDR49A isoform, two TMDs at the C-terminus, both consisting of six transmembrane α-helix segments per TMD were predicted (Fig. 2A). For MDR49B, however, two TMDs at the C-terminus, one consisting of three transmembrane α-helix segments and the other consisting of six transmembrane α-helix segments were predicted (Fig. 2B). Typically, ABC transporters have six predicted membrane-spanning α-helix segments per TMD but the number of segments may vary in each TMD^33^. The predicted proteins for both MDR50 and MDR65 displayed the more typical features of TMD structure, which consisted of six transmembrane α-helix segments per TMD, respectively (Fig. 3A,B).

The location of the three amino acid replacements in each of the three MDR proteins due to non-silent mutations found in *91-R* are given in Figs 2 and 3. T374I, M388L, and E666D for MDR49A; T173I, M187L, and E465D for MDR49B were predicted to be located on the intracellular loop between TMD 1 and TMD 2 for both MDR49A and MDR49B (Fig. 2A,B). For MDR50, T393A was located in the intracellular loop between TMD 1 and TMD 2 and S881F and V1012L were predicted to be in transmembrane segments 7 and 12, respectively (Fig. 3A). For MDR65, K277R was predicted to be in the intracellular loop between transmembrane segment 4 and 5 in TMD 1, I646M in the intracellular loop between TMD 1 and TMD 2, and L992P in the extracellular loop between transmembrane segment 11 and 12 in TMD 2 (Fig. 3B).

### Identification of the alternatively spliced transcripts from *MDR49, 50 and 65*

**Figure 4:**
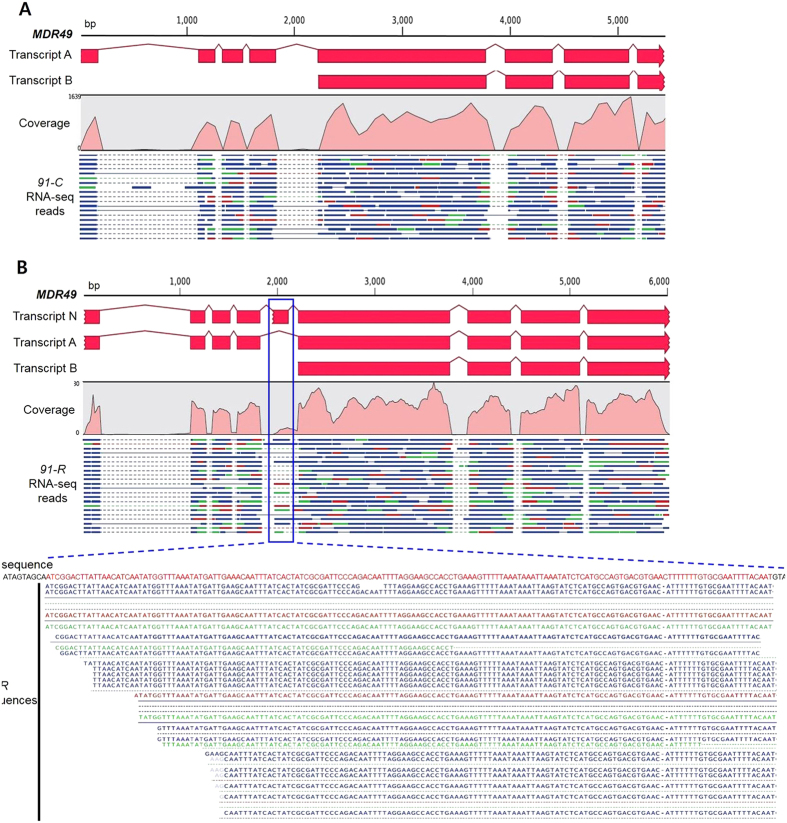
Prediction of *MDR49* alternative splice variants. (**A**) Two alternative splice variants (transcripts A and B) were predicted from *91-C*-*MDR49*. (**B**) Three alternative splice variants (transcripts A, B and N) were predicted from *91-R*-*MDR49*. Optional exon (transcript N) was indicated by blue box from *91-R*-*MDR49*. Several reads uniquely matched to the non-coding RNA region. Black letters represent exonic region; red letters represent non-coding RNA region.

Transcript discovery analysis was performed using RNA-seq data in order to determine whether alternative splicing occurred in these three *MDR* genes when *91-R* and *91-C* were compared. A total average of 157,625,457 raw reads for *91-R* and of 150,788,606 raw reads for *91-C* were obtained, respectively. The average of 3,186 reads for *91-R* and of 4,081 reads for *91-C* were mapped to each MDR gene, respectively (Table S2). Two transcript isoforms had previously been reported in GenBank for *MDR49* (transcripts A and B, Fig. 4). Interestingly, the current analysis of alternative spliced transcript for *MDR49* indicated an additional splice variant in the *91-R* strain. Our analysis found average 145 reads, which matched the non-coding RNA region of *MDR49* and allowed the prediction of an additional exon (transcript N, Fig. 4B). No reads were found matching to this region in *91-C* (Fig. 4A). We confirmed the presence of three alternatively spliced transcripts in *91-R* and two alternatively spliced transcripts in *91-C*. However, the newly found transcript N in *91-R* led to a premature termination of translation. In terms of expression levels between the *MDR49A* and *MDR49B* transcripts, both transcripts were expressed at the same ratio between the *91-R* and *91-C* strains (χ^2^ = 4.626, df = 2, *p* = 0.099) (Table S3).

Unlike *MDR49*, only one transcript variant was found and no differences in alternative splicing were observed for either *MDR50* or *65* when *91-R* was compared with *91-C*. All reads clearly matched to the nine exons in *MDR50* (Supplementary Fig. S3) and to the twelve exons in *MDR65* (Supplementary Fig. S4).

### Effects of three mutations in MDR49A and B from *91-R* on DDT resistance

As *MDR49* was the only gene, of all the *MDR* genes in *Drosophila*, found to be associated with a major effect chromosome interval in the selective sweeps analysis^18^, four transgenic fly strains containing ORFs as follows: (1) *MDR49A* from *91-R* (*91-R*-*MDR49A*); (2) *MDR49B* from *91-R* (*91-R*-*MDR49B*); (3) *MDR49A* from *91-C* (*91-C*-*MDR49A*); and (4) *MDR49B* from *91-C* (*91-C*-*MDR49B*), were generated to investigate this finding in more detail.

**Figure 5:**
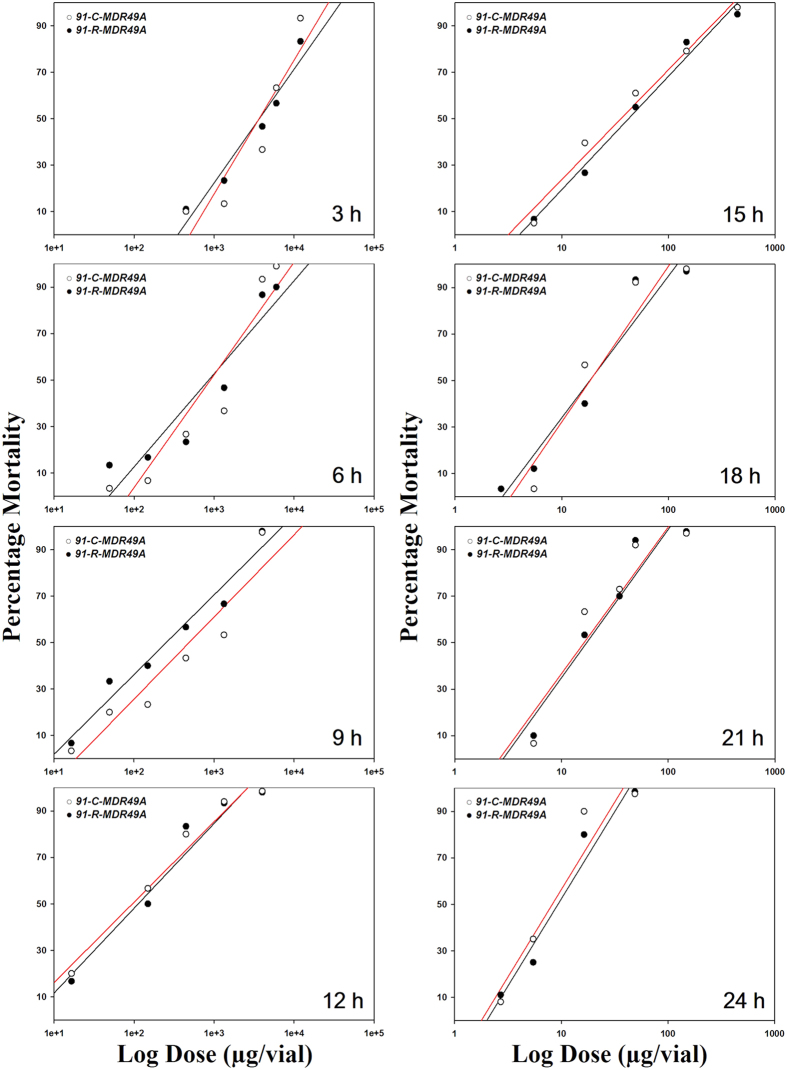
Dose-response curves for the transgenic strains, *91-C*-*MDR49A* and *91-R*-*MDR49A*. Log dose versus percent mortality response curves were determined at different time interval from 3 to 24 h following contact exposure to DDT (μg/vial), respectively. No significant differences between the two transgenic strains were observed at any time interval after DDT treatment.

**Figure 6:**
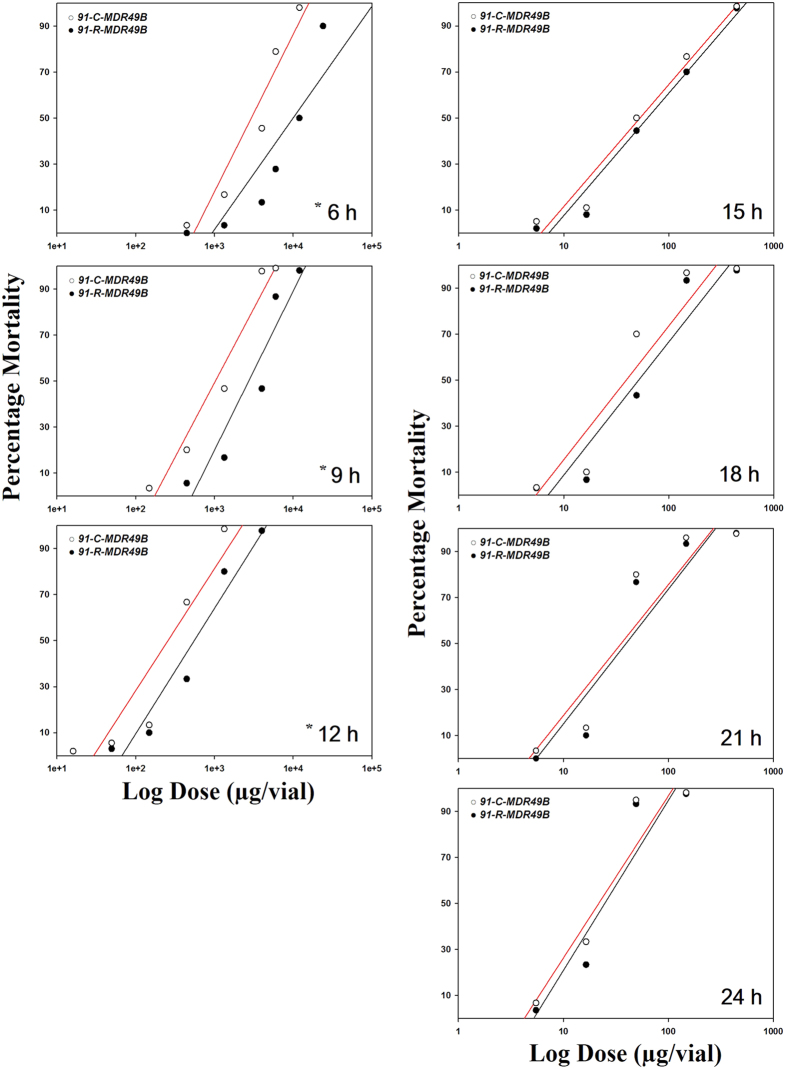
Dose-response curves for the transgenic strains, *91-C*-*MDR49B* and *91-R*-*MDR49B*. Log dose versus percent mortality response curves were determined at different time interval from 6 to 24 h following contact exposure to DDT (μg/vial), respectively. Asterisks indicate statistically significant differences in the dose responses when the two transgenic strains were compared at 6, 9, and 12 h after DDT treatment, as determined by the maximum-log likelihood ratio test (*p* < 0.05).

The susceptibility of these transgenic strains following exposure to DDT is presented in Table 1. When mortality was evaluated at eight different time points (3, 6, 9, 12, 15, 18, 21, and 24 h post-treatment), the test evaluating the hypotheses of equality between the *91-R*-*MDR49A* and *91-C*-*MDR49A* determined that the regression lines were equal (Fig. 5). However, the test evaluating the hypotheses of equality between the *91-R*-*MDR49B* and *91-C*-*MDR49B* determined that regression lines were not equal (Fig. 6). Thus, the LD_50_ values for *91-R*-*MDR49B* were significantly larger than that from the *91-C*-*MDR49B* after 6, 9, and 12 h following DDT treatment and yielded RR of 3.2, 3.0, and 2.1, respectively (Table 1). There were no significant differences in LD_50_ values between two transgenic strains, however, after 12 h of DDT treatment and the test evaluating the hypotheses of equality between *91-R*-*MDR49B* and *91-C*-*MDR49B* from 15 to 24 h of DDT treatment determined that the regression lines were equal (Fig. 6). For 3 h post-treatment, precise estimation of the LD_50_ values for the both *91-R*-*MDR49B* and *91-C*-*MDR49B* were not feasible because its mortality, even at the highest dose examined (50 mg/vial), was still >50%.

Our results support the hypotheses that the *MDR49A* isoform may not be involved in the DDT resistance phenotype, however, the *MDR49B* isoform may play a temporal role in DDT resistance at the early time points of DDT exposure. Furthermore, this finding implies that amino acid alternations in the protein coded for by the *91-R* allele of the *MDR49* gene may be associated with potential resistance mechanisms, including regulating substrate binding affinity at the TMDs or with ATPase activity of the NBDs.

## Discussion

To the authors’ knowledge, the present study represents the first paper demonstrating the combination of amino acid replacements and splice form variants of a *Drosophila* ABC transporter in DDT resistance. Our data demonstrate that one of the two alternative splice forms, *MDR49B*, contributes to DDT resistance, however, the other isoform *MDR49A* does not, in spite of the fact that the resultant respective proteins both contain the same amino acid replacements. Transgenic over-transcription of either *MDR49A* or *MDR49B* obtained from *91-C* did not result in changes in resistance levels to DDT, suggesting that over-transcription of *MDR49* does not represent a mechanism by which *MDR49* plays a role in resistance. These results are in line with previous work by Gellatly and coworkers, which demonstrated a direct relationship between expression levels of *MDR50, MDR65* and *MRP1* and DDT resistance whereas *MDR49* was not over-transcribed in *91-R*^31^.

An additional observation from our RNA-seq data was the occurrence of a third alternative splice variant of *MDR49* from *91-R*. Comparing the additional transcript sequence of *MDR49* with the *Drosophila* genome revealed that the non-coding RNA region generated an optional exon region as a result of alternative splicing with premature stop codon. Thus, this third alternative splice form is unlikely to play any role in the resistance phenotype. Nevertheless, it would be interesting to determine the potential impact of such intense selection pressure by an insecticide upon the prevalence of such “extra” alternative splice forms across the insect genome. One could speculate that the *91-R* strain, under intense DDT selection pressure, acquired an additional transcript, including this optional exon from a non-coding RNA region. Additional research is necessary, however, to confirm this hypothesis. However, the phenomenon of an extra alternative splice form originating from intronic sequence is not without precedent, as previous studies have demonstrated that alternative exons with high homology probably originated from exons that were previously constitutively spliced in order to maintain the ancestral transcript as a major form, whereas alternative exons with low homology probably originated from exonization of intronic sequences^34^.

Nonetheless, previous studies characterized how alternative splicing of a voltage-gated sodium channel in mosquito contributed to insensitivity to pyrethroids, and feasibly indicated a role of splice variants in the pyrethroid resistance phenotype^35^. The current results from the present study imply that *MDR49* may be associated with DDT resistance through a mechanism involving both alternative splicing and amino acid replacements in the protein.

As *MDR49* was the only *MDR* gene previously shown be associated with a major effect chromosome interval in a selective sweep analysis^18^, we tested the hypothesis that this gene was playing a direct role in DDT resistance through the use of DDT bioassays of transgenic *Drosophila* expressing the *MDR49* isoforms from *91-R* and *91-C*. Our results suggest that only the *MDR49B* transcript from *91-R* is involved in DDT resistance and importantly, significant mortality differences were observed only after DDT treatment for 6 h to 12 h intervals while no significant difference was observed after 15–24 h DDT exposure. Thus, the data from this study revealed that structural alteration of the *MDR49* gene provides partial protection from DDT toxicity and suggests that *MDR49B* could play a role during the initial phase of the detoxification process after DDT exposure. Interestingly, MDR proteins are known to translocate exogenous substrates to the outside of the cell using energy obtained from ATP hydrolysis by the ATPase activity associated with the NBDs^36^. In human, two mutations in NBDs disrupt the catalytic activity, causing reduced ATP binding and hydrolysis^37^. Thus, amino acid replacements near NBDs of MDR49B in *91-R* could be directly involved in interrupting the transportation of exogenous substrates like DDT. Furthermore, the role of TMD is to recognize and mediate the passage of substrates across cell membrane. The TMD conformational alteration within MDR49B in *91-R* could enhance the large diversity of substrate specificity and mediate substrate transport.

Previously, Pedra *et al*. (2004) reported that the relative transcript expression of ABC transporter-like gene (CG9892) in the DDT-resistant *91-R* strain was greater than in the DDT-susceptible *Canton-S* strain using whole genome transcript profiles, suggesting a possible association between DDT resistance and ABC transporters^8^. Recently, Gellatly *et al*. (2015) also showed that the ABC-B subgroup (*MDR50, MDR65*) and the ABC-C subgroup (*MRP*1) were over-transcribed in *91-R* when compared to *Canton-S* and using a UAS/RNAi approach showed that knockdown of these genes increased DDT susceptibility in the transgenic flies^31^. Furthermore, the association of over transcription of ABC transporters with insecticide resistance has been confirmed in *Lygus Hesperus*^38^, *Cimex lectularius*^39^, and *Myzus persicae*^40^. Nevertheless, our current findings would suggest that *91-R* strains may use additional mechanisms allowing for DDT resistance besides over expression of ABC transporters. Indeed, the over expression of ABC transporters associated with resistance to insecticides at different time points has been previously reported in *Anopheles stephensi*^41^. The relative expression of an ABC-B subfamily gene was highly upregulated at early time points following permethrin treatment and ABC-G4 was over transcribed at later time points. Also, Atsumi *et al*. (2012) demonstrated that a single amino acid replacement in the second extracellular loop in an ABC transporter gene causes resistance to the Bt toxin *Cry1Ab* in the *Bombyx mori*^42^. To date, however, ABC transporter mutations associated with resistance to insecticides have not been reported in *Drosophila*.

In order to elucidate the actual effects of individual amino acid replacements on ABC transporter activity, a logical next step will be to focus on the heterologous expression of MDR49 using cRNAs, containing these three mutation alone and in all combinations, injected into *Xenopus laevis* oocytes to improve our mechanistic understanding of this novel phase III xenobiotic metabolism reaction involved in DDT resistance in *Drosophila*.

## Methods

### *Drosophila melanogaster* strains

DDT-resistant *91-R* and DDT-susceptible *91-C* strains were obtained from Dr. Ranjan Ganguly (University of Tennessee-Knoxville). Strains were reared on brown diet (Jazz-Mix *Drosophila* Food, Fischer Scientific, Cat. No. AS153) at 25 °C with 8:16 L:D in plastic bottles and transferred to new bottles about every three weeks. Populations of the *91-R* and *91-C* strains have been maintained in the Pittendrigh laboratory for over a dozen years and *91-R* strain has been under continuous selection at DDT concentrations of 100 mg/ml. For the transgenic lines *w*^*1118*^ was used.

### Structure analysis of MDR proteins

Sequence similarity and analysis of protein-specific motifs were performed using BLAST programs on NCBI. Sequence alignments were performed with Clustal Omega (EMBL-European Bioinformatics Institute, Cambridge, UK). The transmembrane domain and membrane topology was predicted with TOPCONS online software (http://topcons.cbr.su.se/)^43^.

### Identification of alternatively spliced transcripts

All RNA-seq databases from *91-R* and *91-C* were imported to the CLC genomic workbench 8.5 software according to the manufacture’s manual (*Qiagen, Valencia, CA, USA*). The “Transcript Discovery” plug-in was used to predict alternative splicing transcripts and genes. Our trimmed reads of each sample with three replications were mapped against *MDR49, MDR50*, and *MDR65 Drosophila* genome sequences extracted from *Drosophila* genome assembly release 6.07 (file dmel-all-chromosome-r6.07.fasta downloaded from Flybase.org) including intergenic regions. The BAM files with mapped reads were deposited to NCBI Short Read Archive (SRA) with the accession number SRP068789. The matched reads for each transcript were visualized in the CLC interface. Each generated transcript was manually examined by comparing RNA-seq reads with the *Drosophila* genomic sequence to identify alternatively spliced variants.

### Cloning of *MDR49* from *91-R* and *91-C* strains for transgenic expression

Two *MDR49* splice isoforms were cloned and sequenced using total RNA from each of the two strains (*91-R* and *91-C*). First-strand cDNA was synthesized by using Superscript™ III reverse transcriptase (Invitrogen, Carlsbad, CA, USA) primed with oligo-d(T). Internal cDNA fragments of the *MDR49A* and *MDR49B* genes were amplified from the first-strand cDNA with a set of gene-specific primers for each strain. The amplified cDNA-specific products were purified using a PCR clean-up kit (Qiagen, CA) and directly sequenced using the gene-specific primers for both ends to cover the full length. All sequences were assembled and compared by using Vector NTI (Invitrogen, CA).

### Transgenic expression of the two *MDR49* splice isoforms in *Drosophila*

The full-length *MDR49A* and *MDR49B* splice isoforms were amplified from the cDNA of both *91-R* and *91-C* strains using Phusion High-Fidelity DNA Polymerase (New England Biolabs, Ipswich, MA). The sequence-specific primer pairs were used (Table S1). The PCR products were purified using the QIAquick PCR Purification Kit (*Qiagen, CA*) and cloned into the pCR2.1 TOPO vector (Invitrogen, CA). Individual clone from both strains were purified with a QIAprep Miniprep kit (*Qiagen, CA*) and sequenced to locate the open reading frame and validate the correct amino acid sequences for *MDR49A* and *MDR49B*. After sequence analysis, the selected clones for both *MDR49* transcripts were sub cloned into the pUAST vector. Transgenic flies were generated by the BestGene Inc (Chino Hills, CA) using the *w*^*1118*^ strain. For the expression of the transgene *MDR49A* and *B*, ubiquitous driver strain (*P*{*w*[*+mC*] *= GAL4-elav.L*}*2*/*CyO*) was obtained from Bloomington *Drosophila* Stock Center (Bloomington, IL). This female ubiquitous driver strain was crossed with our male transgenic strains and selected F1 progeny that showed both GAL4-MDR49A and B by examining wing shape and eye color for mortality bioassay.

### Mortality bioassays

We used a previously described bioassay approach with some modifications^31^. To determine diagnostic doses of DDT, flies were exposed to vials coated a series of concentration of DDT, and mortality was determined after the flies were exposed for 3, 6, 9, 12, 15, 18, 21, and 24 h in the vials. Flies were considered dead when all movement and leg twitching had ceased. The median lethal dose (LD_50_) values and their 95% confidential limits (CLs) were determined by Probit analysis (POLOPC, LeOra Software, Berkeley, CA). Test for the hypotheses of equality (slopes and intercepts are not significantly different) was performed as described by Robertson *et al*. (2007)^44^. The maximum-log likelihood test was used to determine whether the resulting mortality curves from differently treated fly groups were statistically different (*p* < 0.05).

## Additional Information

**How to cite this article**: Seong, K. M. *et al*. Splice form variant and amino acid changes in MDR49 confers DDT resistance in transgenic *Drosophila. Sci. Rep.* **6**, 23355; doi: 10.1038/srep23355 (2016).

## Supplementary Material

Supplementary Information

## Figures and Tables

**Table 1:** Evaluation of DDT toxicity in two DDT-susceptible transgenic lines (*91-C*-*MDR49A* and *91-C*-*MDR49B*) and two DDT-resistant transgenic lines (*91-R*-*MDR49A* and *91-R*-*MDR49B*) of *Drosophila* using a contact exposure to DDT (μg/vial).

Strain	LD_50_^a^ (95% C.L.)^b^				χ^2^(df)				Slope ± SD				RR^c^
	3 h	6 h	9 h	12 h	3 h	6 h	9 h	12 h	3 h	6 h	9 h	12 h	
*91-R-MDR49A*	3,920.3 (2,868.2–5,562)	833.9 (423.9–1,616.4)	241.6 (80.7–719.3)	103.8 (68–154.1)	2.79 (2)	18.43 (2)	17.14 (2)	0.03 (2)	1.6 ± 0.1	1.4 ± 0.1	1.1 ± 0.1	1.4 ± 0.1	1.0^d^ 0.9^e^ 0.5^f^ 1.0^g^
*91-C-MDR49A*	3,893.2 (1,963.2–8862)	971.2 (509.9–1,777.9)	489.1 (164.6–2,251.5)	105.9 (58.6–180.3)					1.9 ± 0.2	2.0 ± 0.1	1.2 ± 0.1	1.4 ± 0.1	
*91-R-MDR49B*	>50,000	11,834.8* (9,603–16,025)	2,986.9* (1,188–5968.5)	600.7* (408–996.6)	–	131.1 (2)	101.1 (2)	36.4 (2)	–	2.1 ± 0.3	2.8 ± 0.2	2.0 ± 0.2	3.2^e^ 3.0^f^ 2.1^g^
*91-C-MDR49B*	>50,000	3,284.4* (1,481–6,398.7)	1,002.3* (438.7–2,192.1)	299.9* (185.6–501.8)					–	2.6 ± 0.2	2.7 ± 0.2	3.0 ± 0.3	
**Strain**	**LD**_**50**_^a^ **(95% C.L.)**^b^				**χ2 (df)**				**Slope** ± **SD**				**RR**^c^
	**15 h**	**18 h**	**21 h**	**24 h**	**15 h**	**18 h**	**21 h**	**24 h**	**15 h**	**18 h**	**21 h**	**24 h**	
*91-R-MDR49A*	39.8 (31.4–50.2)	17.1 (10.9–28.9)	12.2 (7.5–22.7)	7.8 (4.9–14.7)	1.86 (2)	5.1 (2)	3.84 (2)	9.54 (2)	1.7 ± 0.1	2.4 ± 0.2	2.7 ± 0.2	2.6 ± 0.2	1.2^h^ 1.1^i^ 1.0^j^ 1.4^k^
*91-C-MDR49A*	32.3 (12.1–81.7)	15.1 (11.8–19.6)	11.9 (8.4–17.9)	5.7 (5.0–6.6)					1.8 ± 0.1	3.0 ± 0.2	3.3 ± 0.3	2.8 ± 0.2	
*91R-MDR49B*	68.1 (40.5–115.1)	46.2 (40.1–53.2)	35.9 (21.1–58.6)	18.6 (11.9– 29.8)	3.34 (2)	5.19 (2)	1.66 (2)	5.18 (2)	2.8 ± 0.2	3.2 ± 0.3	3.1 ± 0.3	3.7 ± 0.4	1.2^h^ 1.3^i^ 1.1^j^ 1.2^k^
*91-C-MDR49B*	56.8 (37.2–86.2)	36.7 (32.1–42.1)	29.9 (18.4–48.4)	17.9 (11.4–28.7)					2.4 ± 0.2	3.4 ± 0.3	2.9 ± 0.2	3.4 ± 0.3	

^a^Lethal Dose (ug/vial) that killed 50% of the flies.

^b^95% Confidence limit.

^c^Resistance ratio = *91-R* LD_50_/*91-C* LD_50_.

^d^Resistance ratio at 3 h DDT treatment.

^e^Resistance ratio at 6 h DDT treatment.

^f^Resistance ratio at 9 h DDT treatment.

^g^Resistance ratio at 12 h DDT treatment.

^h^Resistance ratio at 15 h DDT treatment.

^i^Resistance ratio at 18 h DDT treatment.

^j^Resistance ratio at 21 h DDT treatment.

^k^Resistance ratio at 24 h DDT treatment.

^*^Log DDT dose versus mortality regressions of *91-R-MDR49A* versus *91-C-MDR49A* and *91-R-MDR49B* versus *91-C-MDR49B* flies were significantly different using the maximum log-likelihood ratio test, respectively (*p* < 0.05).
