# Supplementary material for: Splice form variant and amino acid changes in MDR49 confers DDT resistance in transgenic Drosophila
Source: Sci Rep. 2016 Mar 22;6:23355. doi: 10.1038/srep23355 (PMC4802207; doi:10.1038/srep23355)

Title:

Splice form variant and amino acid changes in MDR49 confers DDT resistance in transgenic *Drosophila*

Keon Mook Seong[a], Weilin Sun[a], John M. Clark[b], Barry R. Pittendrigh[a*]

[a] Department of Entomology, University of Illinois at Urbana-Champaign, Illinois, USA

[b] Department of Veterinary & Animal Science, University of Massachusetts, Amherst, MA, 01003, USA

* Corresponding Author

Barry R. Pittendrigh, Department of Entomology, University of Illinois at Urbana-Champaign, Illinois, USA, pittendr@illinoi.edu

Supplementary table S1. Cloning primers used in this study.

Gene	Sequence	Remarks
Dmel_MDR49A	F: GCAGTATCGCCTCATAGACACAATGG<br>R: TCACTCCAGTTCGCTGTGTTAG	For the cloning of full Dmel_MDR49 ORF
Dmel_MDR49B	F: CCTCATAGACACAATGACCTTTGTG<br>R: TCACTCCAGTTCGCTGTGTTAG	

Supplementary table S2. Summary of RNA-seq data from *91-R* and *91-C*.

Strains	Replicates	Total reads	Genes	Coverages	Mapped reads
91-R	R1	152,800,522	*MDR49A*	137.3	3,270
			MDR49B		2,733
			MDR49N		3,142
			MDR50	92	2,385
			MDR65	80.3	1,499
	R2	152,219,476	*MDR49A*	159.1	3,492
			MDR49B		3,263
			MDR49N		ND
			MDR50	162.2	3,953
			MDR65	84.3	1,696
	R3	167,856,374	*MDR49A*	189.7	4,766
			MDR49B		3,802
			MDR49N		4,607
			MDR50	175.4	4,303
			MDR65	91.3	1,697
91-C	R1	154,166,490	*MDR49A*	265.9	5,414
			MDR49B		5,440
			MDR50	133.7	3,265
			MDR65	112.6	2,523
	R2	142,269,050	*MDR49A*	203.9	4,563
			MDR49B		4,031
			MDR50	176	4,097
			MDR65	98.9	2,234
	R3	155,930,278	*MDR49A*	224.9	5,652
			MDR49B		4,564
			MDR50	208	5,006
			MDR65	117.5	2,178

ND describes ‘not determined’

Supplementary table S3. Comparison of expression level of *MDR49A* and *MDR49B* in *91-R* and *91-C*, based on RNA-seq data.

Gene	Expression value*		ratio**	*p*-value
	91-R	*91-C*		
MDR49A	6.72 ± 0.13	8.01 ± 0.21	0.86	p>0.05
MDR49B	6.67 ± 0.58	6.75 ± 0.32	0.99	

*Expression value was $\log_2$ transformed from raw read coverages.

**Ratio was calculated as *91-R*/*91-C*.

Supplementary figure legend

Supplementary figure S1. Sequence alignment of putative amino acids deduced of the MDR50 proteins from *91-C* and *91-R* strain. Three mutations, T393A, S881F, and V1012L were observed between two strains. The NBDs are homologous for *91-C* and *91-R* strain and each NBD has seven highly conserved motifs. The conserved aromatic, Walker A, Q-loop, ABC signature, Walker B, D-loop, and H-loop are boxed.

Supplementary figure S2. Sequence alignments of the putative amino acids deduced from the MDR65 proteins from the *91-C* and *91-R* strains. Three mutations that resulted in amino acid replacements, K277R, I646M, and L992P, were observed when the *91-R* strain was compared with the *91-C* strain. The NBDs were homologous for both strains and each NBD had seven highly conserved motifs (aromatic, Walker A, Q-loop, ABC signature, Walker B, D-loop, and H-loop), which appear in boxes

Supplementary figure S3. Prediction of the alternative splice variants in *MDR50*. (A) One alternative splice variant was predicted from *91-C-MDR50*. (B) One alternative splice variant was predicted from *91-R-MDR50*.

Supplementary figure S4. Prediction of the alternative splice variants in *MDR65*. (A) One alternative splice variant was predicted from *91-C-MDR65*. (B) One alternative splice variant was predicted from *91-R-MDR65*.

Supplementary figure S1.

```
91-C-MDR50    MTVKNGDVVKDDVNSRSQYKTNIVLGAKLEDSDRDRKSFEPNKSKKKSKHDESDASDEED   60
91-R-MDR50    MTVKNGDVVKDDVNSRSQYKTNIVLGAKLEDSDRDRKSFEPNKSKKKSKHDESDASDEED   60
              ************************************************************

91-C-MDR50    GSQYHEDVKQVSYFQLFRYATKKDRALYVIGLLSAVATGLTTPANSLIFGNLANDMIDLS   120
91-R-MDR50    GSQYHEDVKQVSYFQLFRYATKKDRALYVIGLLSAVATGLTTPANSLIFGNLANDMIDLS   120
              ************************************************************

91-C-MDR50    GLLESGKSYRADDAISTLLLDKVRQFSLQNTYIGIIMLVCSYLSITCFNYAAHSQILTIR   180
91-R-MDR50    GLLESGKSYRADDAISTLLLDKVRQFSLQNTYIGIIMLVCSYLSITCFNYAAHSQILTIR   180
              ************************************************************

91-C-MDR50    SKFFRSILHQDMKWYDFNQSGEVASRMNEDLSKMEDGLAEKVVMFVHYLVAFVGSLVLAF   240
91-R-MDR50    SKFFRSILHQDMKWYDFNQSGEVASRMNEDLSKMEDGLAEKVVMFVHYLVAFVGSLVLAF   240
              ************************************************************

91-C-MDR50    VKGWQLSLVCLTSLPLTFIAMGLVAVATSRLAKKEVTMYAGAAVVAEGALSGIRTVKAFE   300
91-R-MDR50    VKGWQLSLVCLTSLPLTFIAMGLVAVATSRLAKKEVTMYAGAAVVAEGALSGIRTVKAFE   300
              ************************************************************

91-C-MDR50    GEAKEVAAYKERVVAAKILNIKRNMFSGIGFGLLWFFIYASYALAFWYGVGLVIESAYEN   360
91-R-MDR50    GEAKEVAAYKERVVAAKILNIKRNMFSGIGFGLLWFFIYASYALAFWYGVGLVIESAYEN   360
              ************************************************************

91-C-MDR50    YDAGTMITVFFSVMMGSMNIGMAAPYIEAFGITKGACAKVFHIIEQIPEINPIDGEGKKL   420
91-R-MDR50    YDAGTMITVFFSVMMGSMNIGMAAPYIEAFGIAKGACAKVFHIIEQIPEINPIDGEGKKL   420
              ******************************** ***************************
                             Aromatic                     Walker A
91-C-MDR50    NEPLTTIEFKEVEFQYPTRPEVSILNKLNLKIHRGQTVALVGPSGCGKSTCIQLVQRFYD   480
91-R-MDR50    NEPLTTIEFKEVEFQYPTRPEVSILNKLNLKIHRGQTVALVGPSGCGKSTCIQLVQRFYD   480
              ************************************************************
                                           Q-loop
91-C-MDR50    PQAGNLLFNGTNLKDLDINWLRSRIGVVGQEPILFATSIYENIRYGREDATREEIEAAAA   540
91-R-MDR50    PQAGNLLFNGTNLKDLDINWLRSRIGVVGQEPILFATSIYENIRYGREDATREEIEAAAA   540
              ************************************************************
                                       ABC Signature        Walker B  D-loop
91-C-MDR50    AANAAIFIKKLPKGYDTLVGERGAQLSGGQKQRIAIARALIRDPEILLLDEATSALDTAS   600
91-R-MDR50    AANAAIFIKKLPKGYDTLVGERGAQLSGGQKQRIAIARALIRDPEILLLDEATSALDTAS   600
              ************************************************************
                                H-loop
91-C-MDR50    EAKVQAALEKVSAGRTTIIVAHRLSTVRRADRIVVINKGEVVESGTHQELMELKDHYFNL   660
91-R-MDR50    EAKVQAALEKVSAGRTTIIVAHRLSTVRRADRIVVINKGEVVESGTHQELMELKDHYFNL   660
              ************************************************************

91-C-MDR50    VTTQLGEDDGSVLSPTGDIYKNFDIKDEDEEEIKVLSEDEDEDVMVTDEKNKKKKMKKVK   720
91-R-MDR50    VTTQLGEDDGSVLSPTGDIYKNFDIKDEDEEEIKVLSEDEDEDVMVTDEKNKKKKMKKVK   720
              ************************************************************

91-C-MDR50    DPNEVKPMLEVMKMNKPEWLQIAVGCISSVIMGCAMPIFAVLFGSILQILSVKDNDQYVR   780
91-R-MDR50    DPNEVKPMLEVMKMNKPEWLQIAVGCISSVIMGCAMPIFAVLFGSILQILSVKDNDQYVR   780
              ************************************************************

91-C-MDR50    ENSNQYSLYFLIAGIVVGIATFLQIYFFGIAGERLTERLRGLMFEAMLRQEVAWFDDKAN   840
91-R-MDR50    ENSNQYSLYFLIAGIVVGIATFLQIYFFGIAGERLTERLRGLMFEAMLRQEVAWFDDKAN   840
              ************************************************************

91-C-MDR50    GTGSLCARLSGDAAAVQGATGQRIGTIIQSISTLALGIALSMYYEWSLGLVALAFTPFIL   900
91-R-MDR50    GTGSLCARLSGDAAAVQGATGQRIGTIIQSISTLALGIALFMYYEWSLGLVALAFTPFIL   900
              **************************************** *******************

91-C-MDR50    IAFYMQRTLMAKENMGSAKTMENCTKLAVEVVSNIRTVASLGREEMFHQNYIGMLIPAVE   960
91-R-MDR50    IAFYMQRTLMAKENMGSAKTMENCTKLAVEVVSNIRTVASLGREEMFHQNYIGMLIPAVE   960
              ************************************************************

91-C-MDR50    ISKRNTHFRGLVYGLARSLMFFAYAACMYYGTWCVIHRGILFGDVFKVSQAVIMGTASIA   1020
91-R-MDR50    ISKRNTHFRGLVYGLARSLMFFAYAACMYYGTWCVIHRGILFGDVFKVSQALIMGTASIA   1020
              *************************************************** ********

91-C-MDR50    NALAFAPNMQKGVSAAKTIFTFLRRQPSIVDRPGVSRDPWHSEGYVRFDKVKFSYPTRSE   1080
91-R-MDR50    NALAFAPNMQKGVSAAKTIFTFLRRQPSIVDRPGVSRDPWHSEGYVRFDKVKFSYPTRSE   1080
              ************************************************************

91-C-MDR50    IQVLKGLELAVSKGQKIALVGPSGCGKSTCIQLIQRFYDVDEGATLIDECDVRNVSMTNL   1140
91-R-MDR50    IQVLKGLELAVSKGQKIALVGPSGCGKSTCIQLIQRFYDVDEGATLIDECDVRNVSMTNL   1140
              ************************************************************

91-C-MDR50    RNQLGIVSQEPILFDRTIRENISYGDNARNVTDQEIISACKKSNIHEFIANLPLGYDTRM   1200
91-R-MDR50    RNQLGIVSQEPILFDRTIRENISYGDNARNVTDQEIISACKKSNIHEFIANLPLGYDTRM   1200
              ************************************************************

91-C-MDR50    GEKGAQLSGGQKQRIAIARALIRNPKIMLLDEATSALDAESEKVVQDALDAASEGRTTIS   1260
91-R-MDR50    GEKGAQLSGGQKQRIAIARALIRNPKIMLLDEATSALDAESEKVVQDALDAASEGRTTIS   1260
              ************************************************************

91-C-MDR50    IAHRLSTVVHSDVIFVFENGLVCEAGDHKQLLANRGLYYTLYKLQSGAM              1309
91-R-MDR50    IAHRLSTVVHSDVIFVFENGLVCEAGDHKQLLANRGLYYTLYKLQSGAM              1309
              *************************************************
```

Supplementary figure S2.

```
91-C-MDR65    MERDEVSTSSSEGKSQEEAPMAEGLEPTEPIAFLKLFRFSTYGEIGWLFFGFIMCCIKAL    60
91-R-MDR65    MERDEVSTSSSEGKSQEEAPMAEGLEPTEPIAFLKLFRFSTYGEIGWLFFGFIMCCIKAL    60
              ************************************************************

91-C-MDR65    TLPAVVIIYSEFTSMLVDRAMQFGTSSNVHALPLFGGGKTLTNASREENNEALYDDSISY   120
91-R-MDR65    TLPAVVIIYSEFTSMLVDRAMQFGTSSNVHALPLFGGGKTLTNASREENNEALYDDSISY   120
              ************************************************************

91-C-MDR65    GILLTIASVVMFISGIFSVDVFNMVALRQVTRMRIKLFSSVIRQDIGWHDLASKQNFTQS   180
91-R-MDR65    GILLTIASVVMFISGIFSVDVFNMVALRQVTRMRIKLFSSVIRQDIGWHDLASKQNFTQS   180
              ************************************************************

91-C-MDR65    MVDDVEKIRDGISEKVGHFVYLVVGFIITVAISFSYGWKLTLAVSSYIPLVILLNYYVAK   240
91-R-MDR65    MVDDVEKIRDGISEKVGHFVYLVVGFIITVAISFSYGWKLTLAVSSYIPLVILLNYYVAK   240
              ************************************************************

91-C-MDR65    FQGKLTAREQESYAGAGNLAEEILSSIRTVVSFGGEKSEVQRYENFLVPARKASQWKGAF   300
91-R-MDR65    FQGKLTAREQESYAGAGNLAEEILSSIRTVVSFGGERSEVQRYENFLVPARKASQWKGAF   300
              ************************************ ***********************

91-C-MDR65    SGLSDAVLKSMLYLSCAGAFWYGVNLIIDDRNVENKEYTPAILMIAFFGIIVGADNIART   360
91-R-MDR65    SGLSDAVLKSMLYLSCAGAFWYGVNLIIDDRNVENKEYTPAILMIAFFGIIVGADNIART   360
              ************************************************************
                                                                   Aromatic
91-C-MDR65    APFLESFATARGCATNLFKVIDLTSKIDPLSTDGKLLNYGLRGDVEFQDVFFRYPSRPEV   420
91-R-MDR65    APFLESFATARGCATNLFKVIDLTSKIDPLSTDGKLLNYGLRGDVEFQDVFFRYPSRPEV   420
              ************************************************************
                                   Walker A
91-C-MDR65    IVHRGLNIRIRAGQTVALVGSSGCGKSTCVQLLQRFYDPVFGSVLLDDLDIRKYNIQWLR   480
91-R-MDR65    IVHRGLNIRIRAGQTVALVGSSGCGKSTCVQLLQRFYDPVFGSVLLDDLDIRKYNIQWLR   480
              ************************************************************
                   Q-loop
91-C-MDR65    SNIAVVGQEPVLFLGTIAQNISYGKPGATQKEIEAAATQAGAHEFITNLPESYRSMIGER   540
91-R-MDR65    SNIAVVGQEPVLFLGTIAQNISYGKPGATQKEIEAAATQAGAHEFITNLPESYRSMIGER   540
              ************************************************************
                ABC Signature           Walker B  D-loop                   H-loop
91-C-MDR65    GSQLSGGQKQRIAIARALIQNPKILLLDEATSALDYQSEKQVQQALDLASKGRTTIVVSH   600
91-R-MDR65    GSQLSGGQKQRIAIARALIQNPKILLLDEATSALDYQSEKQVQQALDLASKGRTTIVVSH   600
              ************************************************************

91-C-MDR65    RLSAIRGADKIVFIHDGKVLEEGSHDDLMALEGAYYNMVRAGDINIPDEVEKEDSIEDTK   660
91-R-MDR65    RLSAIRGADKIVFIHDGKVLEEGSHDDLMALEGAYYNMVRAGDINMPDEVEKEDSIEDTK   660
              ***************************************************** ******

91-C-MDR65    QKSLALFEKSFETSPLNFEKGQKNSVQFEEPIIKALIKDTNAQSAEAPPEKPNFFRTFSR   720
91-R-MDR65    QKSLALFEKSFETSPLNFEKGQKNSVQFEEPIIKALIKDTNAQSAEAPPEKPNFFRTFSR   720
              ************************************************************

91-C-MDR65    ILQLAKPEWCYLILGTISAVAVGFLYPAFAVIFGEFYAALAEKDPEDALRRTAVLSWACL   780
91-R-MDR65    ILQLAKPEWCYLILGTISAVAVGFLYPAFAVIFGEFYAALAEKDPEDALRRTAVLSWACL   780
              ************************************************************

91-C-MDR65    GLAFLTGLVCFLQTYLFNYAGIWLTTRMRAMTFNAMVNQEVGWFDDENNSVGALSARLSG   840
91-R-MDR65    GLAFLTGLVCFLQTYLFNYAGIWLTTRMRAMTFNAMVNQEVGWFDDENNSVGALSARLSG   840
              ************************************************************

91-C-MDR65    EAVDIQGAIGYPLSGMIQALSNFISSVSVAMYYNWKLALLCLANCPIIVGSVILEAKMMS   900
91-R-MDR65    EAVDIQGAIGYPLSGMIQALSNFISSVSVAMYYNWKLALLCLANCPIIVGSVILEAKMMS   900
              ************************************************************

91-C-MDR65    NAVVREKQVIEEACRIATESITNIRTVAGLRREADVIREYTEEIQRVEVLIRQKLRWRGV   960
91-R-MDR65    NAVVREKQVIEEACRIATESITNIRTVAGLRREADVIREYTEEIQRVEVLIRQKLRWRGV   960
              ************************************************************

91-C-MDR65    LNSTMQASAFFAYAVALCYGGVLVSEGQLLFQDIIKVSETLLYGSMMLAQSLAFTPAFSA  1020
91-R-MDR65    LNSTMQASAFFAYAVALCYGGVLVSEGQLPFQDIIKVSETLLYGSMMLAQSLAFTPAFSA  1020
              **************************** *******************************

91-C-MDR65    ALIAGHRLFQILDRKPKIQSPMGTIKNTLAKQLNLFEGVRYRGIQFRYPTRPDAKILNGL  1080
91-R-MDR65    ALIAGHRLFQILDRKPKIQSPMGTIKNTLAKQLNLFEGVRYRGIQFRYPTRPDAKILNGL  1080
              ************************************************************

91-C-MDR65    DLEVLKGQTVALVGHSGCGKSTCVQLLQRYYDPDEGTIHIDHDDIQHDLTLDGVRTKLGI  1140
91-R-MDR65    DLEVLKGQTVALVGHSGCGKSTCVQLLQRYYDPDEGTIHIDHDDIQHDLTLDGVRTKLGI  1140
              ************************************************************

91-C-MDR65    VSQEPTLFERSIAENIAYGDNRRSVSMVEIIAAAKSANAHSFIISLPNGYDTRMGARGTQ  1200
91-R-MDR65    VSQEPTLFERSIAENIAYGDNRRSVSMVEIIAAAKSANAHSFIISLPNGYDTRMGARGTQ  1200
              ************************************************************

91-C-MDR65    LSGGQKQRIAIARALVRNPKILLLDEATSALDLQSEQLVQQALDTACSGRTCIVIAHRLS  1260
91-R-MDR65    LSGGQKQRIAIARALVRNPKILLLDEATSALDLQSEQLVQQALDTACSGRTCIVIAHRLS  1260
              ************************************************************

91-C-MDR65    TVQNADVICVIQNGQVVEQGNHMQLISQGGIYAKLHKTQKDH                    1302
91-R-MDR65    TVQNADVICVIQNGQVVEQGNHMQLISQGGIYAKLHKTQKDH                    1302
              ******************************************
```

Supplementary figure S3.

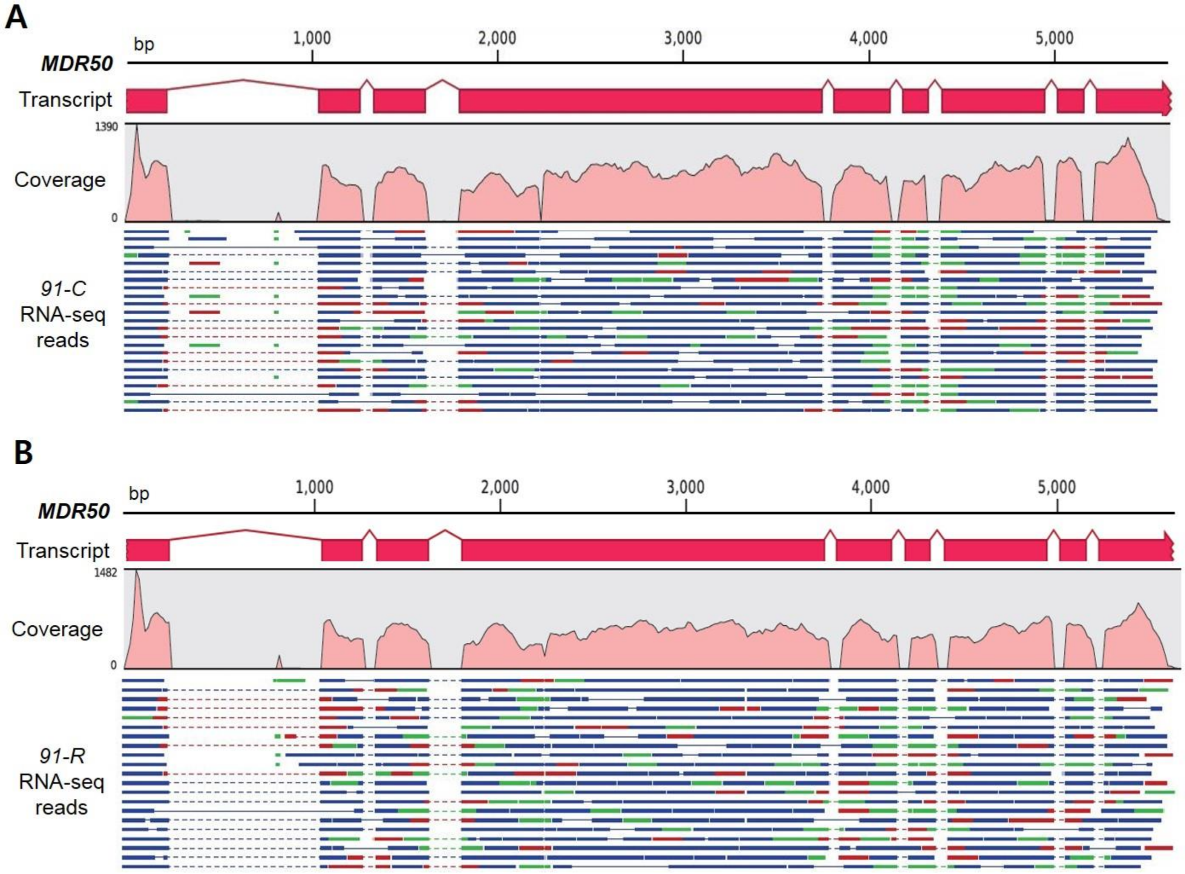

Supplementary figure S4.

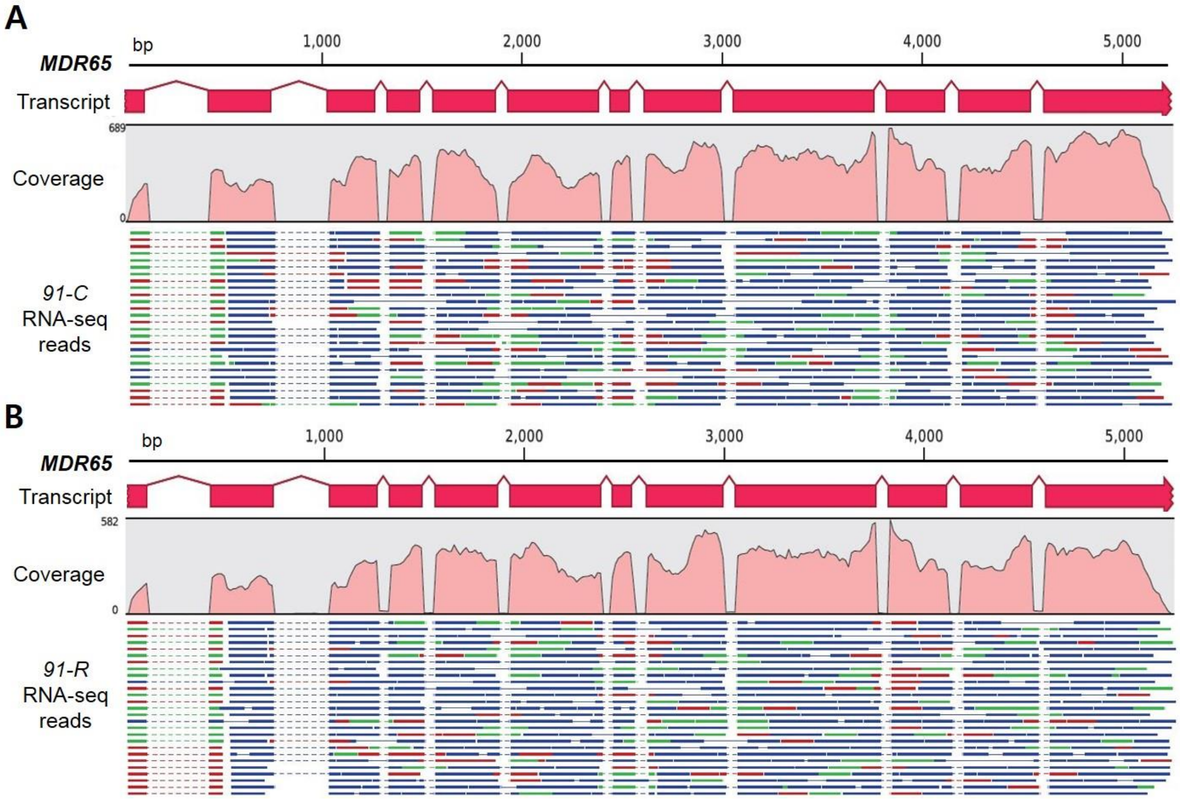

Supplement: Supplementary Information [file srep23355-s1.pdf]
